# Genetic mapping of the *LOSS OF PARTHENOGENESIS* locus in *Pilosella piloselloides* and the evolution of apomixis in the Lactuceae

**DOI:** 10.3389/fpls.2023.1239191

**Published:** 2023-08-25

**Authors:** Ross Bicknell, Marion Gaillard, Andrew Catanach, Robert McGee, Sylvia Erasmuson, Beatrice Fulton, Christopher Winefield

**Affiliations:** ^1^ Department of Breeding and Genomics, The New Zealand Institute for Plant and Food Research Limited, Christchurch, New Zealand; ^2^ Department of Plant and Microbial Biology, University of Zürich, Zurich, Switzerland; ^3^ Department of Plant Science, McGill University, Lincoln, QC, Canada; ^4^ Department of Wine, Food and Molecular Biosciences, Lincoln University, Canterbury, New Zealand

**Keywords:** parthenogenesis (asexual reproduction), apomixis, pilosella, allele sequence divergence, egg cell activation

## Abstract

*Pilosella piloselloides* var. *praealta* (syn. *P. praealta*; *Hieracium praealtum*) is a versatile model used to study gametophytic apomixis. In this system apomixis is controlled by three loci: one that controls the avoidance of meiosis (*LOA*), one that controls the avoidance of fertilization (*LOP*) and a third that controls autonomous endosperm formation (*AutE*). Using a unique polyhaploid mapping approach the *LOP* locus was mapped to a 654 kb genomic interval syntenic to linkage group 8 of *Lactuca sativa*. Polyhaploids form through the gametophytic action of a dominant determinant at *LOP*, so the mapped region represents both a functional and a physical domain for *LOP* in *P. piloselloides*. Allele sequence divergence (ASD) analysis of the *PARTHENOGENESIS* (*PAR)* gene within the *LOP* locus revealed that dominant *PAR* alleles in *Pilosella* remain highly similar across the genus, whilst the recessive alleles are more divergent. A previous report noted that dominant *PAR* alleles in both *Pilosella* and *Taraxacum* are modified by the presence of a class II transposable element (TE) in the promoter of the gene. This observation was confirmed and further extended to the related genus *Hieracium*. Sufficient differences were noted in the structure and location of the TE elements to conclude that TE insertional events had occurred independently in the three genera. Measures of allele crossover amongst the polyhaploids revealed that *P. piloselloides* is an autopolyploid species with tetrasomic inheritance. It was also noted that the dominant determinant of *LOP* in *P. piloselloides* could transmit via a diploid gamete (pollen or egg) but not via a haploid gamete. Using this information, a model is presented of how gametophytic apomixis may have evolved in several members of the Lactuceae, a tribe of the Asteraceae.

## Introduction

*Pilosella piloselloides* var. *praealta* (syn. *P. praealta*; *Hieracium praealtum*) (Asteraceae, Cichorieae) is a model plant used to study gametophytic apomixis, the asexual formation of seeds (Catanach et al., 2006; Rodrigues et al., 2008; Koltunow et al., 2011; Bicknell et al., 2016; Bräuning et al., 2018; Underwood et al., 2022). In this system, apomixis is conferred by the inheritance of dominant determinants at two, unlinked loci: one associated with the avoidance of meiosis (the *LOSS OF APOMEIOSIS* locus; *LOA*) and another with spontaneous embryo formation (the *LOSS OF PARTHENOGENESIS* locus; *LOP*) (Catanach et al., 2006; Koltunow et al., 2011; Shirasawa et al., 2014). In apomictic species, as in almost all angiosperms, embryo maturation requires the support of nutritive endosperm tissue. Apomictic species differ in the origin of this tissue. In some apomicts the endosperm is derived sexually (‘pseudogamous apomixis’) while in others it arises asexually (‘autonomous apomixis’). Apomictic species in the genus *Pilosella* feature autonomous apomixis, the consequence of which is that fertilization is neither required for initiation of the embryo nor for the formation of the endosperm. As a result, seed formation in apomictic *Pilosella* proceeds without pollination and often does so precociously, being well advanced before the flower opens (Koltunow et al., 1998). Autonomous endosperm in *Pilosella piloselloides* var. *praealta* (syn. *H. praealtum*; syn. *Hieracium caespitosum*) was recorded by Catanach et al. (2006) to be associated with the inheritance of a dominant allele at the *LOP* locus. Ogawa et al. (2013), however, described two segregants from amongst the progeny of related *Pilosella* species in which autonomous endosperm was expressed without parthenogenesis, suggesting the presence of an ‘*AUTONOMOUS ENDOSPERM*’ locus (*AutE*) in this genus. The structure, function and location of the *AutE* locus, however, remains unclear.

The dominant actions of the *LOA* and *LOP* loci are inferred by their influence when present as simplex copies. In *Pilosella piloselloides* var. *piloselloides* (syn. *P. piloselloides; Hieracium piloselloides*) and *Pilosella piloselloides* var. *praealta* (syn. *Hieracium praealtum*) the loss of *LOA* by deletion resulted in plants exhibiting a meiotic phenotype, while the loss of *LOP* resulted in plants requiring fertilization to initiate both embryogenesis and endosperm formation (Catanach et al., 2006; Koltunow et al., 2011) The loss of both loci resulted a ‘mutant’ phenotype of sexual reproduction, in this otherwise asexually reproducing plant. The uncovering of sexual reproduction following the removal of these loci supports the proposal that sexual reproduction is the base state with apomixis acting as an overlay process in this species (Koltunow et al., 2011). Further support for this hypothesis comes from studies of gene expression in apomictic and sexual biotypes of *Pilosella*. Tucker et al. (2003) reported that the same genes appear to act in both biotypes throughout most of gametogenesis, indicating that the sexual and apomictic pathways of reproduction in *Pilosella* (syn. *Hieracium*) differ in the expression of only a small percentage of the genes and/or alleles necessary to achieve the formation of a new sporophytic generation. Bräuning et al. (2018) further noted that the transcriptome of an *LOP* mutant of *Pilosella piloselloides* var. *praealta* surveyed over the period of parthenogenesis was substantially similar to that of the corresponding wild-type.

Shirasawa et al. (2014) constructed genetic linkage maps for two apomictic species, *Pilosella piloselloides* var. *piloselloides* (syn. *Hieracium piloselloides*) and *Pilosella piloselloides* var. *praealta* (syn. *Hieracium praealtum*) using SSR markers associated with expressed transcripts. In *Pilosella piloselloides* var. *praealta* the *LOA* and *LOP* loci were mapped to linkage groups LGR15 and LGR08 respectively, supporting previous findings that the two loci are unlinked in this plant. The partial structure of the *LOA* locus was described by Kotani et al. (2014). It was found to be a hemizygous region of low recombination, containing extensive, often complex repeat sequences. This is similar to the reported structure of the apospory-specific genomic region (ASGR) in the apomictic grass species *Pennisetum squamulatum* and *Cenchrus ciliaris*, which has an analogous function to *LOA* and *LOP*, combined (Roche et al., 1999; Akiyama et al., 2004; Zeng et al., 2011). Kotani et al. (2014) addressed the potential role of repeat sequence at the *LOA* locus, noting that *LOA* remained functional when it was separated from the majority of the surrounding repeat sequence through recombination. They concluded that the association between hemizygosity, repeat sequence and apomixis loci is more likely to be a consequence of the action of these loci in suppressing recombination, rather than a requirement for their function.

The complete structure of the *LOP* locus of *Pilosella piloselloides* var. *praealta* is currently unreported, although one gene within the locus, *PAR*, is now known. Underwood et al. (2022) reported the structure and function of the *PAR* gene of the apomict *Taraxacum officinale* (*ToPAR*), and also the orthologous sequence (*PpPAR*) from *Pilosella piloselloides* var. *praealta.* In both systems *PAR* acts as an initiator of parthenogenesis, activating the egg cell to enter into the first mitotic division of embryogenesis. The presence of a transposable element was noted in the promoters of the dominant alleles of *PAR* in both species and it was postulated that this element is responsible for an alteration in the expression of these alleles in apomictic plants.

Apomixis is a form of asexual reproduction. Consequently, it promotes the establishment of uniform populations, and enables the perpetuation of clonal lineages over multiple seedling generations. As with all forms of clonal reproduction, apomixis restricts potential genetic advance through recombination (Van Dijk et al., 2009; Hörandl, 2011; Mau et al., 2015). It has, therefore, been described as an ‘evolutionary dead-end’ (Darlington, 1939; Stebbins, 1950) but this viewpoint is now generally regarded as too simplistic (Van Dijk and Vijverberg, 2005). Apomixis is very seldom expressed as an obligate, or exclusive, trait. Typically, it co-exists with sexual reproduction, both through the perpetuation of populations of inter-fertile, mixed biotypes and/or through the expression of sexual and asexual developmental pathways on the same plant (Matzk et al., 2000; Bicknell et al., 2003; Hörandl and Hojsgaard, 2012; Hand and Koltunow, 2014; Hojsgaard and Hörandl, 2019). In this way ‘facultatively apomictic’ species retain the ability to exploit allelic crossover and recombination, facilitating their advance under selection. The genus *Pilosella* provides a good example. Both sexual and apomictic biotypes are commonly found in close proximity, and they are known to readily hybridize in natural habitats (Krahulcová and Krahulec, 1999; Houliston and Chapman, 2001; Krahulcová et al., 2001; Fehrer et al., 2007; Mráz et al., 2009). Also, apomixis in this genus is facultative, with progeny forming from both sexual and asexual developmental pathways on the same plant, and occasionally from a combination of both Bicknell et al. (2003). One expected consequence of facultative apomixis is that, while allele diversity is expected to occur in these plants, patterns of ‘Allele Sequence Divergence’ (ASD) will differ between genomic intervals associated with the expression of apomixis and regions that are not critical to the inheritance of the trait. This effect arises because alleles which confer elements of apomixis are more likely to reside within clonal lineages. Conversely, alleles that are not involved in the inheritance of apomixis will more frequently reside in sexual biotypes and be subject to greater rates of recombination. Studies of ASD in apomicts, however, are uncommon, principally because so little is known of the loci that control apomixis.

The aims of this study were to; define the limits of the functional domain of the *LOP* locus in *Pilosella piloselloides* var. *praealta*; compare rates of recombination around *LOP* against the physical map of the locus; measure allelic exchange near *LOP* within this autopolyploid species; characterize ASD amongst a group of related species at *LOP*, and finally to measure *LOP* allele exchange through gametes of different ploidy. We propose a model of how gametophytic apomixis may have arisen in the genus *Pilosella* and other members of the Lactuceae.

## Materials and methods

### Plant material

Throughout the study, the accession *Pilosella piloselloides* var. *praealta* R35 was used as the apomictic, wild-type, control. A taxonomic re-assessment of the genera *Hieracium* and *Pilosella* (Bräutigam and Greuter, 2007) resulted in the re-organization of these taxa, and also in name changes for several of the species. In a previous deletion mapping study (Catanach et al., 2006), R35 was given the name *H. caespitosum* C4D. This plant was later reassigned as *Hieracium praealtum* R35 and is now designated as *Pilosella piloselloides* var. *praealta* R35. This is the name used throughout this publication; however, as earlier references use alternative names, synonyms are provided in the introduction and discussion sections to aid understanding. Eleven of the mutants described by Catanach et al. (LOP γ115, LOP γ116, LOP γ136, LOP γ138, LOP γ143, LOP γ144, LOP γ161 and LOP γ179; LOA γ165, LOA γ134 and LOA γ186) were used in the current study to construct tiles of Bacterial Artificial Chromosome (BAC) clones and to facilitate construction of the physical map of *LOP*. Other wild-type plants (as listed in **Table 1**) were obtained from European botanical gardens or were collected from natural habitats.

**Table 1:** Plants used in this study.

	Provenance	Biotype	Ploidy	Unique *PAR* alleles detected
*Pilosella*				
*P. aurantiaca*	Champex, Switzerland (native)	Apomictic	4	3*
*P. cymosa*	Dijon, France (native)	Apomictic	5	5*
*P. glacialis*	Champex, Switzerland (native)	Apomictic	5	4*
*P. lactucella*	Dijon, France (native)	Apomictic	5	5*
*P. officinarum*	Caen, France (native)	Sexual	4	3
*P. officinarum*	Lewis Pass, New Zealand (adventive)	Apomictic	4	4*
*P. officinarum*	Jyväskylä, Finland (native)	Apomictic	4	4*
*P. onegensis*	Parangalitsa, Bulgaria (native)	Sexual	2	2
*P. peleteriana*	Orsieres, Switzerland (native)	Sexual	2	1
*P. piloselloides* var *piloselloides*	Steiermark, Austria (native)	Apomictic	4	4*
*P. piloselloides* var *praealta*	Dijon, France (native)	Apomictic	4	4*
*Hieracium*				
*H. lepidulum*	Twizel, New Zealand (adventive)	Apomictic	5	3*
*H. murorum*	Hanmer, New Zealand (adventive)	Apomictic	5	3*
*H. policheae*	Hanmer, New Zealand (adventive)	Apomictic	5	3*

*A dominant allele at LOP, identified through the presence of a TE in the promoter.

Two polyhaploid populations were produced from R35. Polyhaploids are the product of the parthenogenic development of a reduced egg cell and therefore contain half the genetic material of the maternal plant (Asker and Jerling, 1992). In this case, as the maternal plant was tetraploid at *LOP*, the recovered polyhaploids were diploids. The first population of 500 individuals was produced using the method of Bicknell et al. (2003). Seed from a plant of R35, transgenic for a simplex copy the *codA* negative selectable marker, was sown onto hormone-free tissue culture medium containing 200 mg/L 5-fluorocytosine. Any polyhaploid, lacking the marker gene following meiotic reduction, grew on the selective medium and was recovered. This method provides a simple way of producing polyhaploid populations for mapping studies without significant genome modification, providing the wild-type produces polyhaploids at a favorable rate and the transgene is not in linkage with a genetic region of interest. Approximately 1% of the progeny of R35 are polyhaploids (Bicknell et al., 2003). As 50% of segregants inherit the *codA* marker and do not grow, this method produced polyhaploids at a rate of approximately 0.5% of the seed sown. To provide more polyhaploids, a second method was also used. LOA γ134 is a mutant of R35 with a deletion across the *LOA* locus (Catanach et al., 2006). Consequently, LOA γ134 lacks *LOA* function and produces reduced egg cells following meiosis. Mutant LOA γ134, however, retains the *LOP* locus so egg cells carrying the dominant *LOP* determinant are capable of parthenogenesis, the product of which is a polyhaploid. The mutant LOA γ134 therefore generates polyhaploids at a rate of approximately 50% of the seed sown. A second population of 1,669 polyhaploids was produced by this method, bringing the total number of polyhaploids used in the study to 2,169. All the polyhaploids were tested by flow cytometry to confirm that they were diploids. Data from the two populations were initially analysed separately to be sure that the method of polyhaploid formation did not influence the result, particularly since mutant LOA γ134 was generated via gamma-induced mutagenesis (Catanach et al., 2006). No population effect was seen, so the data are presented collectively. PH70, the polyhaploid plant used for genome sequencing, was derived by the negative selectable marker technique to avoid potential difficulties with mutation. PH70 was chosen for sequencing as: it had dominant determinants at both the *LOA* and *LOP* loci; it formed apomictic seed at a high rate; it was diploid and it was also comparatively vigorous.

For the study of allele sequence divergence genomic DNA was isolated from the following species: *P. aurantiaca*, *P. lactucella*, *P. cymosa*, *P. glacialis*, *P. officinarum*, *P. onegensis*, *P. peleteriana*, *P. piloselloides* var. *piloselloides*, *P. piloselloides* var. *praealta*, *Hieracium lepidulum, Hieracium murorum*, and *Taraxacum officinarum*. The DNA was extracted using a Qiagen DNeasy Plant Mini Kit^®^ in accordance with the supplier’s instructions and normalised to a concentration of 2ng/µL prior to use. For the species *P. officinarum*, four accessions were used, varying in geographic origin and biotype. The ploidy of each plant was determined by flow cytometry, using the staining method of Otto (1990) and measured on a Partec Space flow cytometer, operating Flow Max software (v2.9). Plants of known ploidy were available for the species *P. aurantiaca, P. piloselloides* var. *piloselloides*, *P. piloselloides* var. *praealta* and *P. onegensa.* These were used as ploidy references to estimate the ploidy of the remaining samples (**Table 1**).

### BAC library construction and the identification of seed BACs

A Bacterial Artificial Chromosome (BAC) library of the R35 genome was constructed according to the method of Luo and Wing (2003), utilizing the vector pIndigoBAC536, a derivative of pBeloBAC. The final picked library included 258,048 clones. The average insert size was estimated to be 141kb using 117 randomly selected clones. End sequencing of a random selection of 1,536 clones indicated that the representations of mitochondrial and chloroplastic sequences were 0.13% and 0.33% respectively. The *Pilosella* haploid genome is estimated at 1.9 x 10^9^ bp (http://data.kew.org/cvalues), so the library represents an estimated 21-fold coverage of the haploid genome and a 5.4-fold coverage of the complete genome of the plant R35. The library and printed filters are available from the resource center of the Arizona Genomics Institute under the species name *Hieracium caespitosum* (http://www.genome.arizona.edu).

Secondary digest AFLP (sdAFLP) was conducted using markers described by Catanach et al. (2006) according to the method of Knox and Ellis (2001). The identified bands were used to design primers for seed-BAC identification. BAC clones associated with four *LOP*-linked makers (LOP-516, LOP-93, LOP-110 and LOP-380) were identified and sequenced by Sanger sequencing. Within the BAC library the corresponding BAC addresses are 56L17, 192I22, 501A19 and 289K02, respectively. Linkage to the dominant *LOP* allele was confirmed using a panel of plants (the wild-type R35 and the LOP deletion mutants: γ115, γ116, γ136, γ138, γ143, γ144, γ161 and γ179). The BAC sequences were later used for proofing the genomic assembly and for haplotyping genomic scaffolds.

### Genomic sequencing

DNA was isolated from polyhaploid PH70 using a NucleoMag Plant Kit from Machery-Nagel according to the manufacturer’s instructions. DNA samples were then purified by chloroform (1:1 v/v) extraction using Qiagen MaXtract High Density tubes and concentrated using magnetic bead separation (Kappa HyperPure beads). Twenty micrograms of DNA were size-selected to a lower cutoff of 17kb using a Sage Science Blue Pippin as per the manufacturer’s instructions. Size-separated DNA was concentrated using Kappa Hyper beads and DNA purity, concentration and integrity determined using a Qubit Fluorometer and an Agilent S200 series fragment analyzer.

Genome sequencing was carried out by using the GENEWIZ commercial Next Generation Sequencing service to generate short-read (Illumina) sequencing data, and by Oxford Nanopore MinION sequencing to generate long-read sequence data. Approximately 450GB of raw short-read sequence data (150bp paired-end reads, providing approximately 140x coverage of the diploid genome), were generated. In addition, a further 42x of the diploid genome was generated using ONT Minion sequencing. Sequencing libraries were generated using a LSK-SQK109 sequencing Kit (ONT, UK) and the prepared libraries were sequenced on MIN FLO109 flow cells. Data collection was conducted with ONT’s MinKnow (Oxford Nanopore, Windows version 20.06.5) and base calling of the raw data performed off-line using the NeSI compute cluster and the GUPPY base caller (Oxford Nanopore, LINUX version 4.2.2) using default settings recommended by ONT.

Hybrid assembly of the *Hieracium* genome was carried using the MaSuRCA (Zimin et al., 2013; Luo et al., 2017) and FLYE (Kolmogorov et al., 2019) assembly tools and default conditions. The resulting output files were then used to generate a local BLAST database and screened for homology with *Pilosella* BAC sequences using BLAST (Altschul et al., 1990; Camacho et al., 2009) to identify contigs and scaffolds corresponding to the *LOP* locus.

### Polyhaploid mapping of the *LOP* locus

Polyhaploids arise from the parthenogenic development of meiotically-reduced egg cells and can, therefore, be used to generate uni-parental mapping populations in facultatively apomictic *Pilosella* species (Bicknell et al., 2003; McGee, 2013; Shirasawa et al., 2014). In *Pilosella piloselloides* var. *praealta*, *LOP* acts as a gametophytic locus (Catanach et al., 2006; Koltunow et al., 2011) with the dominant determinant present in every polyhaploid (Koltunow et al., 2011). As a functional copy of *LOP* is required, the functional/structural bounds of the locus can be defined by the limits of recombination within a polyhaploid population. In keeping with other known apomixis loci, the frequency of recombination about *LOP* is low (Shirasawa et al., 2014), so the mapping population needed to be correspondingly large. As mentioned above, 2,169 polyhaploids were used in this study. PCR-based markers were developed, using sequence from the seed-BAC clones, from a syntenic region on LG8 of the lettuce genome (http://lgr.genomecenter.ucdavis.edu) and from the *de novo* sequencing of the PH70 genome. Earlier deletion mapping (Catanach et al., 2006) determined that the LOP locus lay between the flanking markers LOP-516 and LOP-380. Allele-specific markers were developed for LOP-516 and LOP-380 and used to identify a group of 52 polyhaploids recombinant between the flanking markers. Based on synteny to lettuce the genomic interval between these markers, which includes the entire LOP locus, is estimated at 8 Mb. The 52 recombinants were then used to map the limits of LOP to an approximate resolution of 0.15 cM. The primers used in this study are listed in **Table 2**.

**Table 2:** Primers used in this study.

Marker	Distance from PAR (kb)	Forward Primer	Reverse primer
289K02	2,594	AGACCACGCATACGGACACACA	CGGCGGTAGGCCGGTACAAC
Lsat10	1,607	CGAACCGCCTTCCAATAATA	TGCAATCTTTGTGCTGGAGA
445AG	1,401	ACTGATGCAATTACAATCGGCG	TCCTCAATCTAGCATGCTAAGG
606_CT	1,239	TCCAACGACTTTCAAACTTGCG	CGAGGATGCTACTTCTTGGAGG
754_AT	1,092	GGTAGCAAACATCAAATCGCCG	GTACCCGCTATGCTCATCCC
907_CT	940	ACGATTTGGTCCTTATTGTATGCG	TCAGATCCTACCAAATCGCAACC
1110_AG	735	CTCTATCGTGTGCATCCAAAGC	GAGGATCGGTATAATATTTGCATGGG
1123	667	AAGTTGCATTTGTAATGGGGGC	CACGCTTTGAGGCTGATTATGG
1267	667	ATGAAGTTGAACCCGGACTACC	TTTTGTCCCTGCCTATCTCACC
Lsat 46	506	GAGAGAAGATTGCCATCAGAACC	GAATTAAATAGYGGATGGAAGAGG
1405_CT	488	TGACTTAAACCCCGAAATGACC	GCCATCATTTCTTCCATTTCCCC
comp14899	457	TCTTGGCCACCGCCAACAAGGTGA	ACTGGCCCG CGCGGATGTCT
135_T7	398	AATAAAACTGGGTGAATACATGTCCC	GGGATCTATATAGGATTGACAACCCC
cds23	203	CCAGATGAAGTCCAATACGTAATGG	TCCTCACCAGAATATCAGGATCC
135_SP6	198	TTTATAATGAACCTTGTGCAAGTGGG	ACTTGAGCATTTTTAAGGTTTTGACC
335_N12	156	ATCAGCCCAATAAACAACCG	TGTAAAAGACAAATTAAGATGCCAA
Pp.PAR	0	TGACGGTTATTTCTAGCGGTTGG	CACAAAACATAATCCAGTTTCGCG
620_T7	-68	GCTACACTTACAATAAGTTTCTGGGG	ATTTCTTCAAGACCAATTTTACCCCC
comp6390	-506	GGCCCGTGCTGATCGCATTCAAGCGG	TCGTCCCAGTCAGCTGCTCCTTCTTGG
AnnxD3	-1,112	TTCTGGCCCAGCTTGTGATTCCTGCC	GGAGGCAAGCAACTGCTTTTTGTGCCG
398_O14	-1,175	TTACACCACCGTCTTCATTG	GTCTCGCTAAACCGAACACT
501	-1,417	GGACCTTAGATGGAATTCGCA	CAAGTTGTCGAAAACCTTCCG
EIF3e	Approx -5,000,000	ATGATAATGGAAGAACACAAATCATTG	ATGATAATGGAAGAACACAAATCATTG
56_L17	Approx -6,000,000	ACCTCTTCCTCGTCCCGAAAGGT	GCAGGGTGCTGATTGTAGATACTCCG

### Patterns of allele inheritance

Two linked genes were selected to determine patterns of homologue exchange close to the *LOP* locus of *P. piloselloides* var. *praealta*: *Annexin D3* (*PpAnnD3*) which is located 1.1Mb from the *PAR* gene and *Eukaryotic translation Initiation Factor* 3e (*PpeIF3e*) which is located approximately a further 4Mb from *PAR* (estimate based on synteny with lettuce) (Reyes-Chin-Wo et al., 2017). Using sequence from cloned AFLP markers 110 and 93 (Catanach et al., 2006), BACs were identified that included alleles of these genes linked to the dominant allele of the *PAR* gene. Redundant primers and low-stringency hybridization were then used to develop intron-spanning allele-specific markers, for all four alleles of *PpAnnD3* and *PpeIF3e.*

Allele segregation was then assessed on a population of 287 polyhaploids, generated by the marker segregation approach and 109 from the deletion mutant approach. For each diploid plant the two alleles at *PpAnnD3* and *PpeIF3e* were scored and the rates of recombination calculated. Alleles in linkage with the dominant determinant at *PpPAR* were designated as ‘Allele 1’. As the *PpPAR* allele is gametophytic in action and necessary for polyhaploid formation, it was present in all polyhaploids. Alleles in linkage with *LOP-PAR*, therefore, were also commonly present and easily identified. The remaining alleles for each gene were arbitrarily designated as ‘Allele 2, 3 and 4’.

### Allele sequence divergence at *PAR*

The *PARTHENOGENESIS* (*PpPAR*) gene is located within the *LOP* locus of *P. piloselloides* var. *praealta* (Underwood et al., 2022)*. PAR* encodes a K2-2 zinc finger protein, known to be necessary for the expression of parthenogenesis in the related genus *Taraxacum* (Underwood et al., 2022). Using alignments with published genomes, the genomic sequence of PH70 and data from the MinIon sequencing of *Hieracium policheae* genomic DNA, primers were designed that amplified exonic sequence for this gene in a range of plant species. The sequences of the primers used are listed in **Table 2**. Established ribosomal ITS primers (Gardes and Bruns, 1993) were also used to phylogenetically order the species under study.

For each species/accession, amplicons for *PAR* and rRNA ITS region were separately generated, barcoded, normalized for concentration, combined and sequenced using the Oxford nanopore MinION sequencing platform. The sequenced amplicons were then processed using the Geneious Prime software suite (Version 2022.0.1) For each gene/species combination at least 2,000 reads were used. Consensus allele sequences were derived using the ‘*De Novo* Assembly’ function and proof-checked by eye using the read alignment tool. Tree assemblies were conducted with the ‘Geneious Tree Builder’ tool using a HKY genetic distance model, a cost matrix of 65% similarity and bootstrap resampling with 5,000 replicates. Published sequence for *Lactuca sativa* was used to provide an outgroup. For each of the apomictic species studied, the dominant allele at *PpPAR* was readily identifiable, due to the presence of transposon-derived sequence in the promoter of the gene, as described by Underwood et al. (2022). Only *PAR* coding sequence was considered in the analysis.

### Allele inheritance in haploid and diploid gametes

As described above, the deletion mutant LOA γ134 has a deletion across the dominant *LOA* allele associated with apomeiosis (genotype */loa/loa/loa). Therefore, LOA γ134 only produces reduced egg cells following the action of meiosis (Catanach et al., 2006). Egg cells carrying the dominant *LOP* allele can then give rise to a diploid seedling through the action of parthenogenesis (a polyhaploid). As LOA γ134 is simplex for the dominant *LOP* allele, all its polyhaploids are heterozygous for *LOP* (*LOP*/*lop*) and they are also meiotic (*loa*/*loa* or *loa/*-), (see **Table 3**). We therefore reasoned that the polyhaploids of LOA γ134 should, in turn, predominantly form haploid egg cells through the action of meiosis. Any egg cell inheriting the dominant *LOP* allele might then be capable of development into a haploid seedling, provided the dominant *LOP* allele was capable of transmission through a haploid female gamete and/or that haploid plant formation was possible with only the dominant *LOP* allele present. A total of 200 polyhaploids were tested. For each polyhaploid, seed was collected from at least 20 capitula, sown onto hormone-free tissue culture medium and maintained under sterile conditions to ensure the survival of weak plants. Germinated seedlings were ploidy tested using a Partec Space flow cytometer and the *PpPAR* alleles were determined using primers listed in **Table 2**. Similarly, to test allele transfer through haploid male gametes, 25 polyhaploids were crossed, as the pollen parent, with a sexual, diploid accession of *Pilosella onegensis*. For each cross-combination, 15 capitula were tested and the seed sown in tissue culture.

**Table 3:** Allele inheritance through gametes of different ploidy.

Seed Plant	Genotype^1^ [Ploidy]	Treatment	Plants^2^ tested	Plants forming viable seed	Seedlings^3^ recovered	Seedling genotype [Ploidy]
LOA γ134^4^	*loa/loa/loa/*^ [4x] *LOP/lop/lop/lop*	No pollination	1	NA	1,669	*loa/-* [2x] *LOP/lop*
Polyhaploids of LOA γ134	*loa/-* [2x] *LOP/lop*	No pollination	200	10	23	0: (*loa; LOP)* [1x] 22: (*loa/-; LOP/lop)* [2x] 1: (*loa/loa/-; LOP/lop/lop)* [3x]
Polyhaploids of LOA γ134	*loa/-* [2x] *LOP/lop*	Crossed as a pollen donor onto sexual *P. onegensis*	25	0	0	NA

1 Genotype of the seed parent at the LOA and LOP loci. ^ A deleted allele. - Either a recessive allele or a deleted allele.

2 Twenty capitula were tested for each polyhaploid genotype.

3 Total across all plants.

4 LOA γ134 is a tetraploid deletion mutant lacking the dominant LOA allele (hence it is triploid for LOA).NA, Not Applicable.

## Results

### The *Pilosella* genome

Genomic sequence of the diploid apomict *Pilosella piloselloides* var. *praealta* PH70, assembled using the hybrid assembler MaSuRCA (Zimin et al., 2013; Luo et al., 2017) resolved the diploid genome into 21,000 contigs, with a predicted haploid genome size of 1.6 Gbp. Analysis of the completeness of the genome gave a BUSCO score of 94%. To identify and interrogate the *LOP* locus, a BLAST library was prepared from these contigs in GeneiousPrime Version 2023.1.1 (www.geneious.com). Sequences of predicted genes, marker sequences and BAC sequences were used to identify contigs with suitable sequence identity to sequences previously associated with the *LOP* locus. To resolve haplotigs associated with the dominant and recessive alleles of *LOP*, BAC sequences and marker sequences were used to manually assemble and resolve individual contigs across 1.6 Mbp. All genome and amplicon sequences are available under the BioProject accession code: *PRJNA982544: Pilosella piloselloides Genome sequencing and assembly (TaxID: 1628030).*

### Size of the LOP locus in Pilosella piloselloides var. praealta

**Figure 1:**
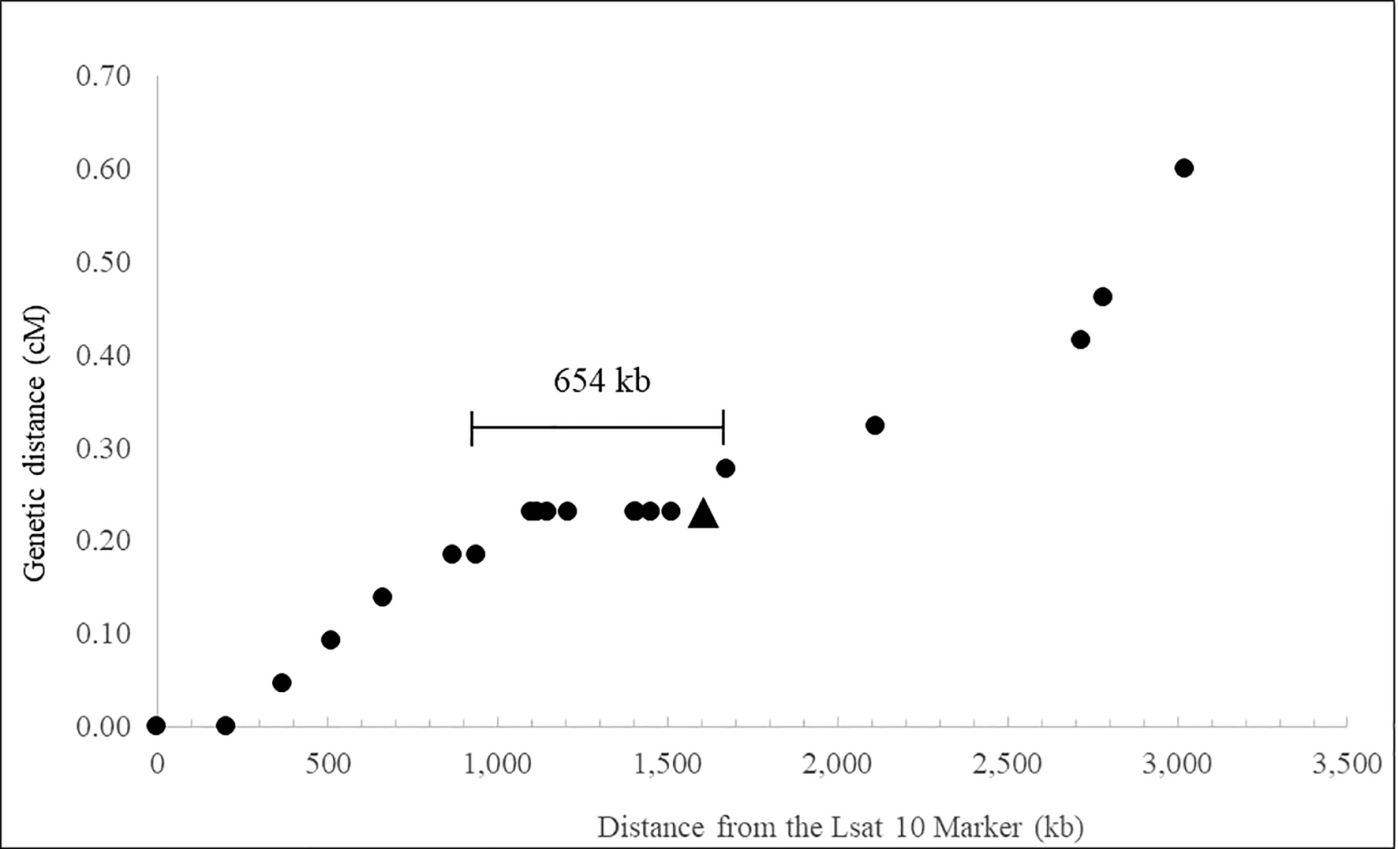
Recombination at the *LOP* locus of *Pilosella piloselloides.* ● A SCAR or SSR marker. ▲ The *PpPAR* gene.

**Figure 1** illustrates the relationship between the physical and recombination-based maps of the *LOP* locus of *P. piloselloides* var. *praealta* PH70, approximately centered on the *PpPAR* gene and extending over a total genomic interval of approximately 3 Mb. The illustrated physical distances are based primarily on dominant *LOP* allele sequence, resolved by combining the outputs of the MaSuRCA (Zimin et al., 2013) and FLYE (Kolmogorov et al., 2019) assembly tools. Estimations of genetic distance are also based on a single recombinant class (those inheriting the dominant *LOP* allele) and the total population size (2,169 polyhaploids) is based only on plants which inherited that allele, as polyhaploids do not form without it. Consequently, both the physical and genetic maps presented in **Figure 1** represent the dominant allele of the *LOP* locus in *P. piloselloides* var. *praealta*.

Polyhaploid mapping defined the functional limits of the *LOP* locus to a region between markers LOP_1267 and 620_T7, a genomic interval of 654 kb (**Figure 1**). Deletion mutant LOP γ138, which lacks *LOP* function, has a break point within this interval, suggesting that the *LOP* locus may be as small as 527 kb. Alternatively, this result may indicate that *LOP* includes more than one gene critical to its function. As previously noted (Underwood et al., 2022) the *PAR* gene lies within the mapped interval and mutant LOP γ138 lacks *LOP* function (Catanach et al., 2006). The total genetic distance of the 3 Mbp genomic region studied was estimated as 0.6 cM.

### Patterns of allele inheritance

**Table 4** records the observed distribution of allelic inheritance, for two loci: *Annexin D3* (*PpAnnD3*), which is located 1.1 Mb from the *PAR* gene, and *Eukaryotic translation Initiation Factor* 3e (*PpeIF3e*), which is located a further 4 Mb from *PAR*, in a population of 287 polyhaploids. All 287 polyhaploids carried the *PpAnnD3*-1 allele, which is tightly linked to the dominant *LOP* locus, but they varied with respect to the other allele present. No significant difference was observed between the frequency of inheritance of alleles *PpAnnD3-*2 and *PpAnnD3-*3 (χ^2^ p > 0.1); however, a highly significant bias against the inheritance of allele *PpAnnD3-*4 was apparent (χ^2^ p < 0.001). Crossover events between the loci detected all possible allele combinations, except between alleles 1 and 2.

**Table 4:** Allelic linkage in a population of 287 polyhaploids.

Allele	*PpEIF3e*-1*	*PpEIF3e*-2	*PpEIF3e*-3	*PpEIF3e*-4	Total (%)
*PpAnnD3*-1*	280	0	4	3	**287 (100)**
*PpAnnD3*-2	0	120	0	1	**121 (42)**
*PpAnnD3*-3	0	1	127	0	**128 (45)**
*PpAnnD3*-4	0	1	1	36	**38 (13)**

*Allele in linkage with the dominant PpPAR allele at the LOP locus.Gray shaded areas remain in original linkage.

### Phylogenetic relationships

**Figure 2:**
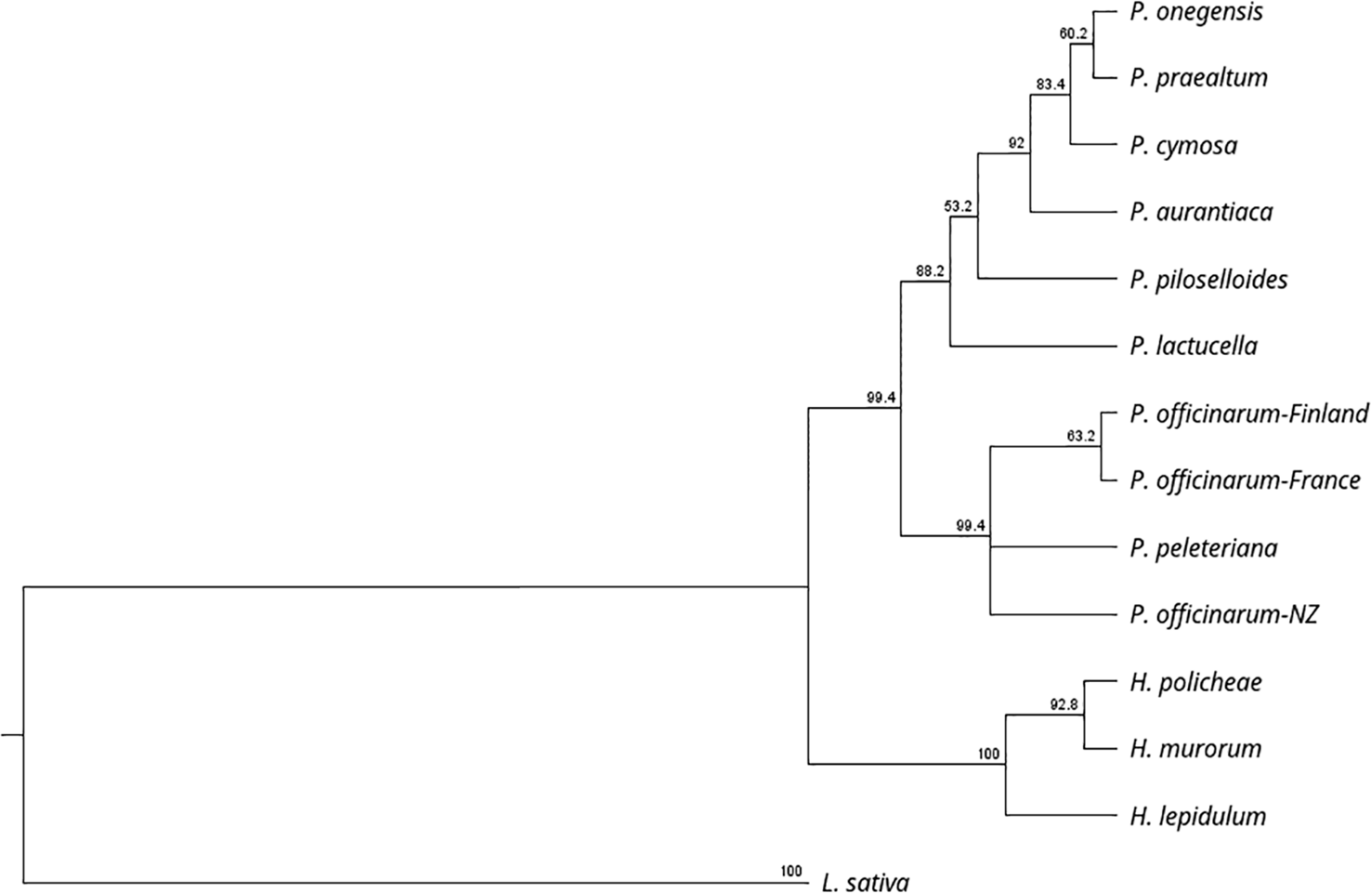
rRNA sequence diversity using ITS primers.

**Table 1** lists the plants used in this study, their biotype, ploidy, and the numbers of unique allelic sequences at *PAR* determined by our analysis. For some of the accessions the number of unique allelic sequences equaled that predicted from the ploidy analysis; however, in many cases a lesser number was determined. It is unclear whether this was due to similarities between alleles and/or whether it resulted from differential-PCR amplification because of differences in primer specificity. **Figure 2** illustrates the taxonomic relationship of the plants studied, as defined by rRNA-ITS sequence, with the inclusion of *Lactuca sativa* as a related outgroup. Species within the two, closely related genera, *Pilosella* and *Hieracium*, grouped into distinct clades, in accordance with their reported taxonomic relationship (Bräutigam and Greuter, 2007).

### Allele sequence divergence at *PAR*

**Figure 3:**
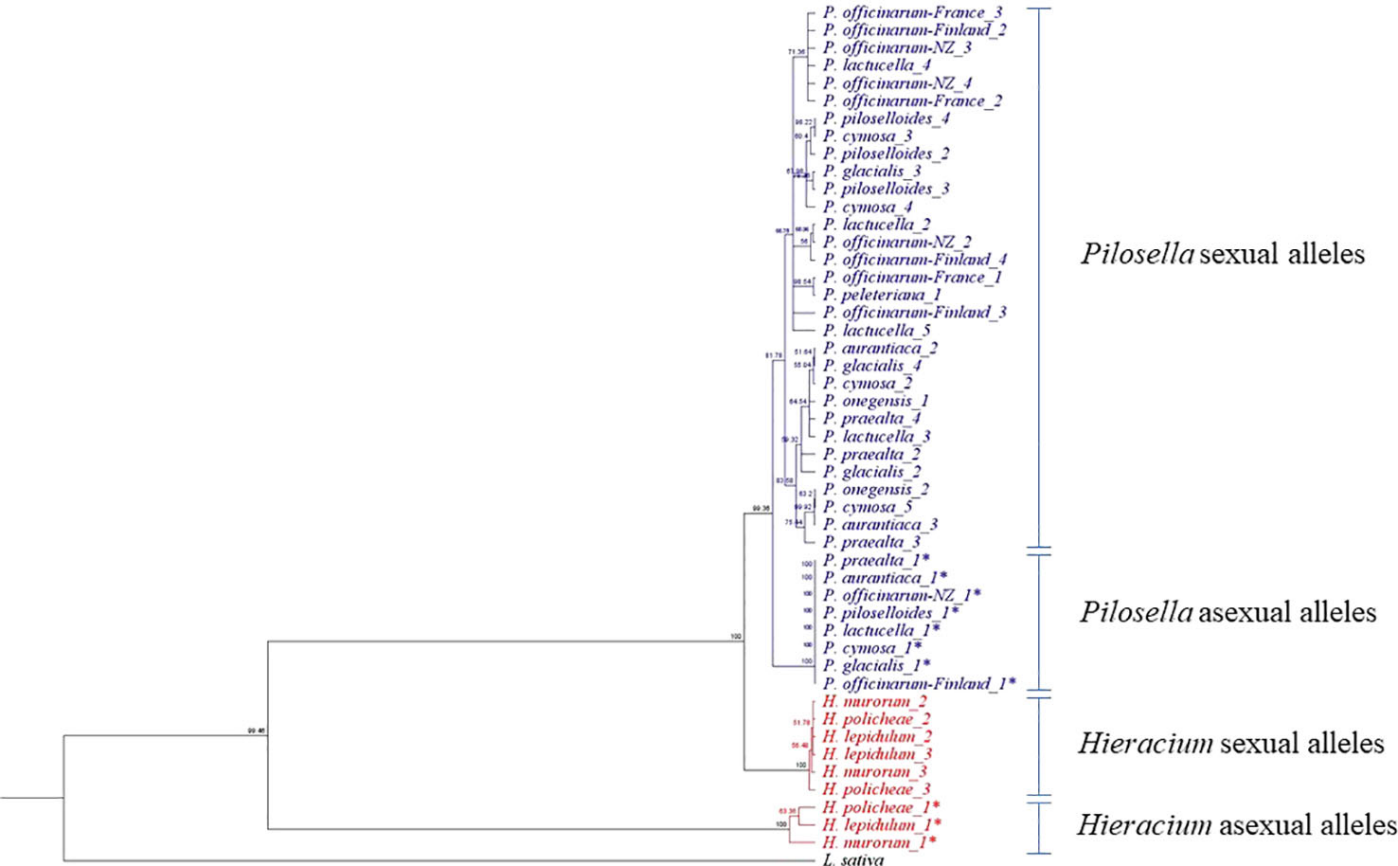
Allele Diversity at *PAR*. * A dominant allele, identified by the presence of a TE in the promoter.

The cladogram of allele sequence relationships at the *PAR* gene is illustrated in **Figure 3**. As observed with rRNA, sequences from the two genera, *Pilosella* and *Hieracium*, clearly separated. Dominant alleles at *PAR*, which associate with the expression of parthenogenesis (labeled PAR-1*) were identified in the three apomictic accessions of *Hieracium* and the nine apomictic accessions of *Pilosella* studied. For *Pilosella*, dominant *PAR* alleles clustered into a single, invariate clade. Dominant alleles in the *Hieracium* species also clustered but were more variable than those of *Pilosella*.

### Structure of dominant *PAR* alleles

**Table 5** lists the observed characteristics of dominant *PAR* alleles in *Hieracium policheae*, *Pilosella piloselloides* and *Taraxacum officinale*. In all cases a class II transposable element was present, positioned in the promoter of *PAR* within a conserved 200 bp interval, previously described by Underwood et al. (2022). The location, length, terminal repeat sequences, TE superfamily and estimated age of the insertion events, however, differed significantly between the genera studied. The presented Ks values and predicted ages of allele separation are based solely on *PAR* exonic sequence and therefore need to be considered with caution. *PAR* is a gene that resides in a region of suppressed recombination and appears to be under strong stabilizing selection. The presented values, therefore, are best viewed in relation to each other rather than as absolute estimates of the age of genomic divergence.

**Table 5:** Characteristics of *PAR* alleles.

Genus	Mechanism of apomixis	Ks (s.e.m)	Est. age (MYA)^1^		Upstream transposon		
				Insertion site^3^	Terminal repeat sequence^4^	Family^5^	Length
*Hieraciu6^5^*	Mitotic Diplospory	0.017 (0.008)	1.06	-154	GAGTAAATTACAAAAATGGTCCCTGTGGTTATATATAGTTTTTGAATCAAGTCCAGTTTTTTCAATTGT GAGTAAATTACAGAAATGGTCCCTATGGTTATATG^A^TTTTTTGAATCAAGTCCAGATTTTTTAATTGT	Mu	1,233
*Pilosella^7^*	Apospory	0.014 (0.004)	0.87	-137^2^	ATGAAAAACTATCGGACCGGCCCTG ACGAAAAACTAATGGACCGGCCCTG	hAT	1,282
*Taraxacum^8^*	Meiotic Diplospory	0.084 (0.012)^2^	5.23^2^	-110^2^	CAGGGCCGGCCA CAGAGCCGGCCC	hAT	1,335

1 Estimated age of dominant/recessive allele divergence at PAR.

2 Published values (Underwood et al., 2022).

3 Position relative to the start codon of PAR.

4 Alignment of the LHS and RHS reverse complement. Mis-aligned bases are highlighted. ^ A missing base.

5 Transposon superfamily based on the host target site duplication (Wicker et al., 2007).

6 Based on Hieracium policheae sequence.

7 Based on Pilosella piloselloides sequence.

8 Based on Taraxacum officinale sequence.

### Allele inheritance in haploid and diploid gametes

**Table 3** records the frequency, ploidy and genotype of progeny derived from the use of LOA γ134 and its polyhaploid progeny. The genotype of LOA γ134, with respect to the *LOP* locus, is *LOP/lop/lop/lop*. The polyhaploid progeny of that plant, therefore, were expected to have the genotype *LOP/lop*, as was observed (**Table 3**). Using similar reasoning, the diploid polyhaploids were expected to produce haploid progeny by parthenogenesis with the genotype *LOP*; however, none was recovered, despite the large number of plants tested. Similarly, no evidence was seen of any haploid pollen function in the diploid polyhaploids plants. Interestingly, viable seedlings were recovered from ten of the polyhaploids. Of the 23 seedlings recovered, 22 were diploid and heterozygous for *LOP* and one was triploid and heterozygous for *LOP*.

## Discussion

### *LOP* maps to a 654 kb interval

Shirasawa et al. (2014) constructed a genetic map for the accession *P. piloselloides* var. *praealta* R35, positioning 418 markers into 18 linkage groups. The location of the *LOP* locus, however, could not be defined for this accession but was defined in a study using a smaller population segregating for *LOP* transferred from the closely related parental line D36 (*Pilosella piloselloides* var. *piloselloides*). In that population *LOP* mapped to a single linkage group, but only to within a region with low marker density. In the current study, using polyhaploid mapping, the *LOP* locus of *P. piloselloides* var. *praealta* was mapped to a 654 kb genomic interval syntenic to linkage group 8 of *Lactuca sativa* (Reyes-Chin-Wo et al., 2017) with a mapping resolution of approximately 0.15 cM (**Figure 1**). The *LOP* locus is, therefore, significantly smaller than the analogous ‘Apospory Specific Genomic Region’ (ASGR) of the apomictic grass *Pennisetum squamulatum* and closely related *Cenchrus ciliaris* (Sherwood et al., 1994; Ozias-Akins et al., 1998). The ASGR is estimated to be approximately 50 Mbp in size and is believed to be composed largely of repeat sequence (Ozias-Akins et al., 1993; Ozias-Akins et al., 1998; Roche et al., 2001; Roche et al., 2002).

Polyhaploids are known to form in *P. piloselloides* var. *praealta* through the gametophytic action of *LOP* (Bicknell et al., 2003; Koltunow et al., 2011). The mapped region, therefore, represents both a functional and a physical domain for *LOP.* Whether the bounds of the mapped region are defined by suppressed recombination and/or due to the presence of more than one critical element within the locus remains unclear. The deletion mutant LOP γ138, which lacks *LOP* function (Catanach et al., 2006), has a break point within the 654 kb interval. If the bounds of the locus are defined by suppressed recombination alone, then this suggests that the *LOP* locus may be as small as 527 kb. However, as described above the phenotype of LOP γ138 could also be the result of a partial loss of *LOP* functionality. The *PpPAR* gene, which has been identified as a critical determinant in the expression of parthenogenesis in *P. piloselloides* var. *praealta* (Underwood et al., 2022) maps within the 654kb interval (**Figure 1**) but is positioned 68kb from a recombinant flanking marker, placing it very near the edge of the mapped interval. This suggests that either a mechanism is acting to suppress recombination on only one side of *PAR*, or that other genes or regulatory elements are present in the remaining non-recombinant region of the *LOP* locus which are necessary for the formation of polyhaploids through parthenogenesis.

### Tetrasomic allelic exchange indicates an autoploid origin for *P. piloselloides* var. *praealta R35*

The accession *P. piloselloides* var. *praealta* R35 is tetraploid at the *LOP* locus, as evidenced by flow cytometric analysis (**Table 4**) and the presence of four distinct allelic sequences amongst the genes sequenced. Tetraploid plants can arise either through the duplication of a single genome (auto-polyploidization) or by the fusion of two distinct species, followed by genome duplication (allo-polyploidization) (Harlan and De Wet, 1975; Ramsey and Schemske, 2002; Ranney, 2006). Tetrasomic patterns of crossover, where allelic exchange can occur between any of the four alleles present, is characteristic of autopolyploidy, whilst disomic inheritance, where an allele will exchange with only one other, is characteristic of allopolyploidy. **Table 4** clearly illustrates the action of tetrasomic exchange in the formation of polyhaploids amongst the progeny of R35, indicating that this an autopolyploid species.

Because of the gametophytic action of *LOP*, every polyhaploid inherited allele 1 (linked to the dominant allele at *PAR*) but polyhaploids differed in the second allele present. Although alleles 2 and 3 were similarly represented in the polyhaploid population, there was a significant bias against the inheritance of allele 4. Evidence of crossover between allele 4 and the remaining alleles (**Table 4**) indicates similarity in sequence, yet allele 4 was clearly either less frequently transmitted through the egg cell following meiosis and/or the subsequent survival of the allele 1: allele 4 genotype was disadvantaged. Either possibility indicates that recessive (sexual) allele inheritance at *LOP* is influenced by selection and, therefore, that recessive alleles play a functional role in this system. Underwood et al. (2022) proposed such a mechanism for alleles at the *PAR* locus of *Taraxacum* and *Pilosella*, hypothesizing that recessive alleles may direct expression of the gene in the pollen grain, while the dominant allele directs expression in the egg cell, placing both under natural selection. Our data suggest that recessive *par* alleles are also critical to the development and/or function of the egg cell as well.

### Allele inheritance in haploid and diploid gametes

It is reported that dominant factors governing gametophytic apomixis seldom, if ever, transmit through haploid gametes, yet they will transmit through diploid or polyploid gametes (Asker and Jerling, 1992; Van Dijk et al., 2009). Nogler (1984) appears to have been the first to have noted this phenomenon, following a study of the pollen-mediated transmission of apomixis in *Ranunculus auricomus*. He proposed that an ‘apomixis’ allele may be haploid-lethal, requiring the co-inheritance of another, complementing ‘sexual’ allele to ensure transfer. An expected consequence of this mechanism would be to establish apomixis in polyploid lineages, as is commonly observed (Nogler, 1986). Underwood et al. (2022) proposed that dominant and recessive alleles of the *PAR* gene, the dominant allele of which confers parthenogenesis in *Taraxacum* and *Pilosella*, may be expressed in different tissues of the flower. If both functions are essential for female gametophyte development and/or function, then this would meet the conditions of Nogler’s hypothesis, limiting transmission of the dominant allele to heterozygotic gametes. Similarly, Van Dijk et al. (2009) demonstrated haploid pollen lethality was linked to the *DIP* locus of the apomict *Taraxacum officinale*. The dominant *DIP* allele confers apomeiosis in this system, which is a separate component of apomixis from parthenogenesis. It is intriguing, therefore, to note that both apomeiosis and parthenogenesis-conferring alleles appear to confer haploid lethality and can, therefore, only transmit in gametes also carrying at least one recessive allele.

In the current study the *LOP* locus of *Pilosella piloselloides* var. *praealta* transmitted readily through the diploid egg cells of the plant LOA γ134 but not through the haploid egg cells of the diploid derivatives of LOA γ134. Furthermore, the pollen of the polyhaploids also appeared to be non-functional. We conclude, therefore, that the dominant allele of *LOP* cannot transmit through either female or male haploid gametes in this system, in agreement with Nogler’s hypothesis. The failure of all pollen function, however, suggests that pollen malfunction may be a general problem with these diploid plants, probably associated with the greater extent of exposed genetic load in polyhaploids. Interestingly, 22 diploid seedlings and one triploid seedling were recovered from the diploid polyhaploid plants tested (**Table 3**). The triploid plant was probably the product of self-fertilization involving the union of a reduced and an unreduced gamete. All the diploid seedlings were heterozygous for *LOP*, indicating that they were either derived following self-fertilization and or through the action of apomixis. The expression of apomixis in these plants, however, would require a mechanism for apomeiosis, yet the *LOA* locus is known to be absent in this plant material. This finding will be further discussed below, with respect to its implications for the evolution of apomixis in this system.

### The structure of *PAR* and allelic sequence divergence at this locus

Genomic regions subject to reduced amounts of homologous recombination are known to accumulate mutational differences, including the accumulation of sequence derived from transposable elements (Kimura et al., 1963; Muller, 1964; Corral et al., 2009). As a consequence, these loci tend to increase in size over time and the allelic sequences of genes within them become increasingly divergent. This effect has been recorded for specific genomic regions within individuals (such as the sex chromosomes of mammals) and at the population level between related sexual and clonal lineages (Mark Welch and Meselson, 2000). The presence of ASD has, therefore, been proposed as an indicator of ancestral asexuality (Corral et al., 2009). As described above, the *LOP* locus of *Pilosella* is a genomic region of suppressed recombination (**Figure 1**) and the perpetuation of apomictic lineages would have further isolated genomes from the influences of meiotic recombination. We, therefore, reasoned that ASD was likely to be measurable in this system, and that the pattern of ASD might inform our understanding of the evolution of apomixis in this plant group. The genus *Pilosella* is a member of the Lactuceae, a tribe of the Asteraceae. *Pilosella* is closely related to the genus *Hieracium* (Bräutigam and Greuter, 2007) and more distantly related to the comprehensively characterized genera *Lactuca* (lettuce) and *Cichorium* (chicory) (Tutin et al., 1976). Most species and clones of *Pilosella* are polyploid, facultative, gametophytic apomicts, using the mechanism of apospory to generate clonal seeds. During apospory, an unreduced embryo sac arises from a nucellar cell, alongside similar meiotically-derived structures within the ovule. Competition between the meiotic and apo-meiotic structures leads to the formation of a mixture of sexually and asexually derived seed. Typically, asexually derived seed predominates. In *P. aurantiaca* (syn. *H. aurantiacum*) and *P. piloselloides* var. *piloselloides* (syn. *H. piloselloides*) meiotically derived seed was recorded at rates of 2.6% and 2.8% respectively (Bicknell et al., 2003). Furthermore, obligate sexual accessions are known to occur in some *Pilosella* species (Gadella, 1972; Gadella, 1984; Gadella, 1991) including *P. officinarum* (as used in this study), and entirely obligate sexual species are also known, such as the diploid *P. onegensis.* For *Pilosella*, therefore, sexual reproduction continues to operate and to contribute to the formation of seed in most plants and in most populations, albeit often at a low rate.

In contrast, plants in the genus *Hieracium* are almost entirely polyploid, obligate gametophytic apomicts, generating seeds by mitotic diplospory of the Antennaria type (Crane, 2001; Mráz et al., 2009; Mráz and Zdvořák, 2018). In this form of gametophytic apomixis, an unreduced embryo sac forms directly from a nucellus cell without the mediation of any meiotic event. Meiotic products, therefore, do not form in the ovule, as seen during apospory, so the resulting seed is solely of clonal origin (Asker and Jerling, 1992). Sexual biotypes of *Hieracium* are known, but amongst the hundreds of described species they appear to be restricted to a small number of diploids of restricted distribution (Mráz and Paule, 2006; Mráz et al., 2011; Mráz and Zdvořák, 2018).

**Figure 2** illustrates the taxonomic relationship between *Pilosella* and *Hieracium*, as defined by rRNA ITS sequence diversity. It supports a recent separation of *Pilosella* and *Hieracium* into two distinct clades (Bräutigam and Greuter, 2007) and illustrates their association with the closely related model species *Lactuca sativa*. **Figure 3** illustrates allelic diversity for the *PAR* coding sequence in the accessions studied. As detailed above, *PAR* lies within the region of suppressed recombination that defines the *LOP* locus. The separation of *Pilosella* and *Hieracium* is again clearly evident in **Figure 3**, which includes the dominant *PAR* alleles, suggesting they have different origins, as confirmed by sequence analysis of the transposon insertion sites and internal transposon sequence (**Table 5**). Notably, the dominant *PAR* alleles of *Pilosella* cluster into a distinct clade, separately from the recessive alleles of the individual accessions tested. This indicates that the dominant alleles share a common and recent origin, supporting the theory that apomixis can radiate rapidly through pollen transfer of the enabling alleles (Van Dijk et al., 2009; Hojsgaard and Hörandl, 2019).

### The expression of apomixis in the Lactuceae

Gametophytic apomixis is a comparatively common trait in the Lactuceae. Of the 70 genera assigned to this tribe, seven (*Taraxacum, Hieracium, Pilosella, Ixeris, Chrondrilla, Cichorium and Crepis*) are known to include apomictic species (Noyes, 2007). An incidence rate of 10% of genera is well above the estimated rate amongst all flowering plant genera of 0.8% (Carman, 1997). The factor(s) that predispose members of this taxon to the expression of gametophytic apomixis are incompletely understood, but available data indicate that apomixis evolved from sexual reproduction many times in this group, rather than being co-inherited from a common apomictic ancestor. One line of evidence is the diversity of apomictic mechanisms found in the Lactuceae. Of the genera listed above, two (*Crepis* and *Pilosella*) form asexual seeds by apospory, three (*Chondrilla*, *Ixeris* and *Taraxacum*) by meiotic diplospory, and one (*Hieracium*) by mitotic diplospory (Carman, 1997; Crane, 2001; Noyes, 2007). The defining differences between these mechanisms relate to events within the ovule at the time of meiosis. Unlike sexual plants, apomictic species either avoid, or utilize a truncated form of meiosis to generate a cell competent to enter gametogenesis, the subsequent product of which is an unreduced embryo sac (Asker and Jerling, 1992). The collective term for the avoidance of the reduction/division step of meiosis in apomicts is ‘apomeiosis’. As apospory, meiotic diplospory and mitotic diplospory differ significantly in the developmental processes involved in apomeiosis, it appears likely that they are controlled by different genetic elements.

Conversely, all the apomictic members of the Lactuceae spontaneously initiate both embryogenesis (parthenogenesis) and endosperm formation (autonomous endospermy) without pollination, indicating a possible commonality in these component processes of apomixis across the Lactuceae. This conclusion was recently proposed by Underwood et al. (2022), who reported that the gene *PAR* was a key determinant in the expression of parthenogenesis in both *T. officinale* and *P. piloselloides* and that the dominant (asexual) alleles conferring parthenogenesis differed from the corresponding recessive (sexual) alleles by the presence of a TE in a highly conserved 200 bp region of the *PAR* promoter. Critically, the insertion site, length and internal sequence of the TE’s were different, implying that they arose from independent insertion events. *Taraxacum officinale* uses meiotic diplospory to form asexual seeds, while *P. piloselloides* uses apospory. In the current study, this observation was further extended to include *Hieracium policheae*, a species using mitotic diplospory, the third mechanism of gametophytic apomixis found in the Lactuceae. Again, the *PAR* gene was found to be modified by a non-autonomous type II transposon, the insertion site of which also located to the conserved promoter region of *PAR* but differed from those of *T. officinale* and *P. piloselloides* (**Table 5**). Furthermore, analysis of synonymous SNP variation also indicates that the *Taraxacum* insertion event occurred earlier than the *Hieracium* and *Pilosella* events (**Table 5**).

### Model for the evolution of apomixis in the Lactuceae

Gametophytic apomixis in flowering plants requires three key elements: apomeiosis, parthenogenesis, and the development of an endosperm to ensure the nourishment of the developing embryo. Endosperm formation in apomicts often occurs by a sexual process, but apomeiosis and parthenogenesis represent significant departures from sexuality. Notably, both apomeiosis and parthenogenesis act as deleterious traits when operating alone. Apomeiosis, when expressed without parthenogenesis, leads to the formation of unreduced eggs that require fertilization to initiate embryogenesis. Fertilization then leads to an increase in ploidy with each generation and ultimately to the isolation of the trait in genotypes with reduced vegetative fitness and reduced fertility (Bicknell and Koltunow, 2004). Conversely, parthenogenesis, when expressed without apomeiosis, leads to the production of progeny with successively decreasing ploidy, also resulting in reduced fitness and lost fertility. There is, therefore, a question as to how gametophytic apomixis evolved in seven genera of the Lactuceae, when it requires the coordinated establishment of two independently deleterious traits.

**Figure 4:**
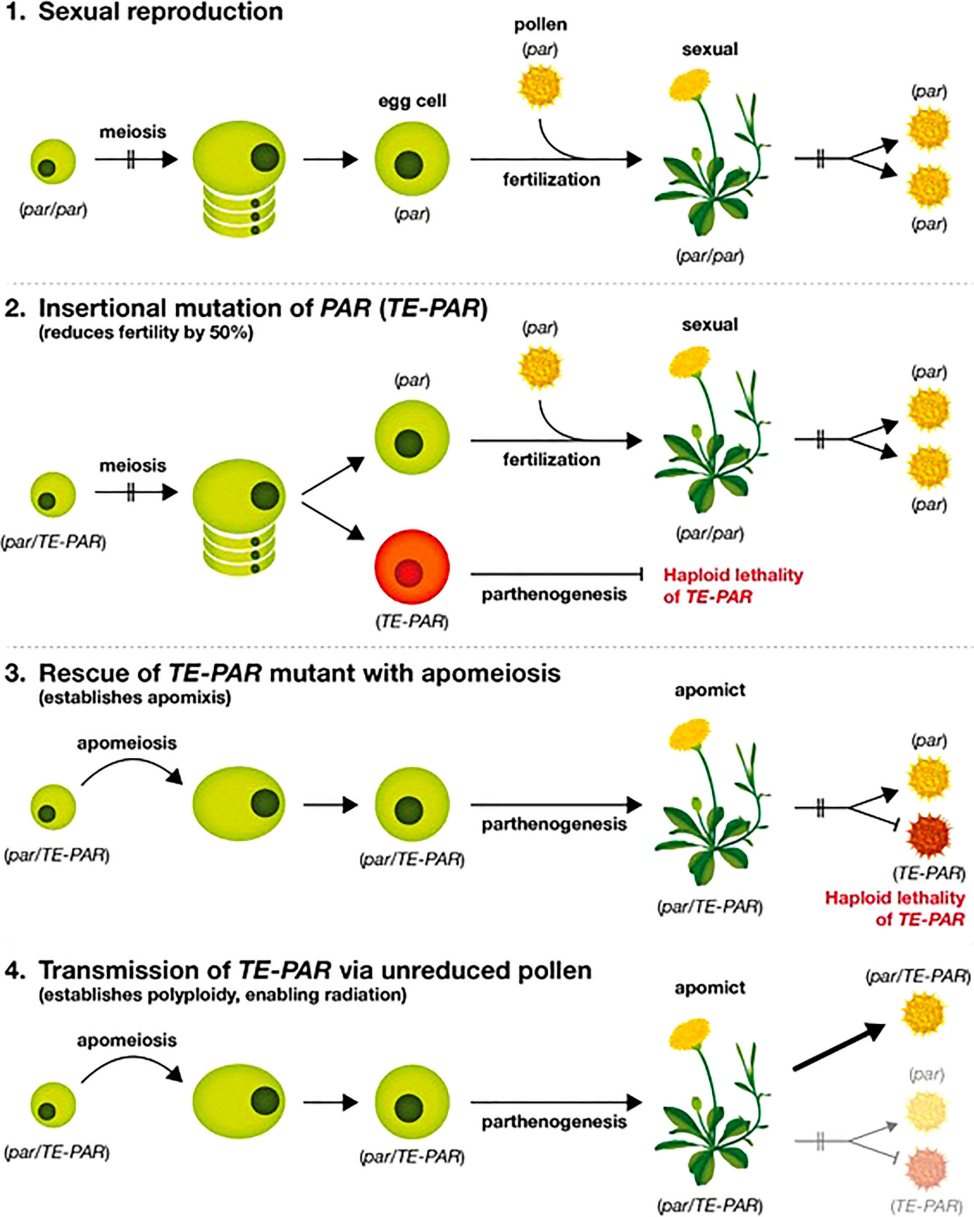
Model for the evolution of apomixis in the Lactuceae.

Based on the data presented above and on observations within the literature we present a model for the evolution of gametophytic apomixis in the Lactuceae (**Figure 4**). We propose that the primary event in the evolution of apomixis is the insertion of a transposable element within a 200 bp conserved region of the *PAR* gene promoter, immediately upstream of the start codon (Underwood et al., 2022) as illustrated in step 2 of **Figure 4**. This establishes a dominant allele for *PAR*, the expression of which promotes spontaneous egg cell activation without fertilization (parthenogenesis) as described by Underwood et al. (2022). We have evidence that this event occurred independently in at least three separate apomictic genera within the Lactuceae: *Taraxacum*, *Pilosella* and *Hieracium* (Underwood et al., 2022) (see also **Table 5**, **Figure 3**). Given the differences noted between these elements (**Table 5**), it appears that the precise identity of the transposon is not critical, however the three elements do share a number of common characteristics that may be of importance. All are class II, DNA-mediated elements, although they differ in their internal sequence, terminal repeat sequence and TE superfamily. All are non-autonomous elements, and they all have imperfectly matched terminal repeat sequences that are likely to prevent their excision. Also, although they all resemble MITE elements in their structure (Wicker et al., 2007), they are all longer than a typical MITE element. Notably, they are all of a similar length (1,233 – 1,335 bp) indicating either the possible presence of regulatory sequences within them or the critical separation of regulatory elements within the native promoter of *PAR*, either of which may direct the ectopic expression of *PAR*.

Meiosis will remain functional following such a TE insertion event, therefore 50% of gametes will carry this modified allele and 50% will not. Our data suggest that *PAR* alleles modified by a TE insertion cannot transmit through haploid gametes, either via eggs or pollen (**Table 3**) so an immediate outcome of the *TE*-*PAR* mutation would be to reduce both seed and pollen fertility by 50%. While this would restrain the spread of the *TE*-*PAR* mutation, it is unlikely to prevent it altogether, as unreduced pollen grains are known to form in a wide variety of flowering plant species (Brownfield and Köhler, 2011). The mediation of unreduced pollen promotes the formation of polyploid progeny, so it is interesting to note that gametophytic apomixis is almost exclusively found in polyploid plants, often existing alongside diploid, sexual relatives (Asker and Jerling, 1992). At this step in the evolution of apomixis, therefore, modified *PAR* alleles would be restricted either to single individuals or to a limited number of polyploid (probably triploid) offspring derived from outcrossing. We predict, therefore, that most *TE*-*PAR* mutations are lost before a more durable form of apomixis is achieved.

If, however, a mechanism of apomeiosis subsequently evolves, it would act to ‘rescue’ a *TE*-*PAR* mutation by enabling reproduction through apomixis rather than sexual reproduction (step 3 in **Figure 4**). Such mutations are likely to occur rarely, so it is notable that gametophytic apomixis is almost exclusively associated with a perennial habit; it is often seen in plants with other forms of vegetative increase; and it typically occurs in polycarpic species that produce abundant seed (Asker and Jerling, 1992). These are all traits that increase the numbers of gametes produced and/or extend the life of a clone, increasing the likelihood that a form of apomeiosis will establish before a modified *TE*-*PAR* allele is lost. In the Lactuceae, for example, apomicts are restricted to perennial species, whilst annuals such as lettuce (*Lactuca sativa*) are well represented amongst the sexual members of the group. The form of newly installed apomeiosis does not appear to be critical for the rescue of dominant *PAR* alleles, as apospory, meiotic diplospory and mitotic diplospory are all found in closely related genera within the Lactuceae. It is also notable that, in the two cases studied: diplospory in *Taraxacum* (Vijverberg et al., 2004) and apospory in *Pilosella* (Koltunow et al., 2011), apomeiosis is conferred by dominant alleles, which are the most likely to establish apomixis in a mutant background where *TE-PAR* alleles are not participating in fertilization.

Finally, the collective instalment of apomeiosis and parthenogenesis would only lead to the perpetuation of a single clone, unless there was a mechanism enabling the dispersal of the critical alleles into a wider germplasm pool. Pollen transfer is the most likely mechanism to mediate this as apomixis restricts access to the female gamete. As described above, dominant alleles conferring both apomeiosis and parthenogenesis appear to transfer only in diploid or polyploid gametes, so the mediation of unreduced pollen grains is the most likely mechanism for their transfer. Unreduced pollen promotes the production of polyploid progeny following the fertilization of a sexual recipient (step 4 in **Figure 4**). In this way polyploid apomictic clones would rapidly establish and hybridize, facilitating their diversification and adaptation over time as previously proposed (Van Dijk et al., 2009; Hojsgaard and Hörandl, 2019). This process, presumably, underlies the observation that the apomicts of *Pilosella* are autopolyploid species (**Table 4**) and that the dominant alleles of *Pilosella* share a very high degree of similarity (**Figure 3**), indicating a common origin and recent exchange between interfertile plants within this genus.

The evolution of the remaining component of apomixis, endosperm formation, remains unclear. Apomicts in the Asteraceae typically develop an endosperm through the spontaneous fusion and subsequent division of the polar nuclei (‘autonomous endosperm’). Conversely, most apomicts beyond the Asteraceae develop an endosperm following the fertilization of polar nuclei, in a manner similar to sexual seed formation (‘pseudogamous apomixis’) (Asker and Jerling, 1992; Crane, 2001). This suggests a predisposition in the Asteraceae towards autonomous endosperm formation, but the nature of that predisposition remains speculative. In *Pilosella piloselloides* var. *praealta* R35, autonomous endosperm formation was lost when the *LOP* locus was deleted (Catanach et al., 2006), indicating that it is either encoded by genetic elements within the *LOP* locus and/or that it requires other genes but is dependent on the action of *LOP* to be expressed. Ogawa et al. (2013) however, reported the genetic uncoupling of autonomous endosperm formation from both apospory and parthenogenesis in *Pilosella piloselloides* var. *piloselloides* (syn. *Hieracium piloselloides* D3), a relative of *Pilosella piloselloides* var. *praealta* R35. This indicates the action of another locus (*AutE*) controlling autonomous endosperm and therefore a further evolutionary step in the development of apomixis in this system, but the location and structure of the *AutE* locus are unknown.

## Conclusions

The *LOSS OF PARTHENOGENESIS* (*LOP*) locus, which mediates spontaneous embryo formation in *P. piloselloides*, acts as a non-recombinant genomic interval with an estimated size of 654 kb. The *PAR* gene lies near a limit of that interval, within 68 kb of the nearest recombinant marker. Beyond the *LOP* locus, allelic exchange occurs between all four alleles in this system, indicating tetrasomic inheritance in an autopolyploid system. Allele sequence divergence was assessed for the *PAR* genes of *Pilosella* and *Hieracium.* In *Pilosella*, dominant *PAR* alleles appear to share a recent co-ancestry, whilst recessive alleles clustered in a manner suggestive of reticulate exchange between species in the genus. In *Hieracium*, dominant alleles also clustered separately to the recessive alleles, but both were well separated from the *Pilosella* clades, indicating a separate origin. Further evidence of this separation came from sequence analysis of the *Hieracium* dominant alleles. Whilst both *Hieracium* and *Pilosella* dominant *PAR* alleles have a transposon insertion immediately upstream of the *PAR* start codon, the length, location, terminal repeat and internal sequences of the different transposons differed significantly, indicating they represent separate ancestral mutations of *PAR*. The importance of ploidy on *LOP* allele transfer was then tested. Dominant ‘parthenogenesis’ alleles of *LOP* could only transfer through egg cells, when they were accompanied by a recessive *lop* allele. Haploid transfer of a dominant *LOP* allele was never observed, neither through the egg cell nor pollen. Interestingly, biased inheritance was observed between the recessive alleles present, indicating a functional role for both dominant and recessive alleles in reproduction in *Pilosella*. Using the information outlined above, a model for the establishment of apomixis in the Lactuceae is presented.

## Data availability statement

The datasets presented in this study can be found in online repositories. The names of the repository/repositories and accession number(s) can be found below: https://www.ncbi.nlm.nih.gov/, BioProject PRJNA982544.

## Author contributions

RB, MG, AC, CW: Contributed to the research plan. RB, MG, AC, RM, SE, BF, CW: Contributed to the data collection. RB, MG, AC, RM, SE, BF, CW: Contributed to writing the manuscript. All authors contributed to the article and approved the submitted version.
